# Spatial Variation in Mycosphere Soil Bacterial Communities of *Leucopaxillus giganteus* and Its Environmental Drivers in Shanxi, China

**DOI:** 10.3390/microorganisms14092029

**Published:** 2026-09-11

**Authors:** Zhen Li, Ruixuan Wu, Mingqin Luo, Yang Xiao, Fei Yu

**Affiliations:** Shanxi Key Laboratory of Efficient Cultivation of Forest Resources, College of Forestry, Shanxi Agricultural University, Jinzhong 030801, China; lizhen@sxau.edu.cn (Z.L.);

**Keywords:** *Leucopaxillus giganteus*, geographic variation, mycosphere soil, soil bacterial community

## Abstract

*Leucopaxillus giganteus*, a wild saprotrophic fungus with high edible and medicinal value, has not yet achieved large-scale artificial cultivation. To elucidate the abiotic and biotic factors governing its growth, we analyzed physicochemical properties and bacterial communities of mycosphere and bulk soils from Shanxi Province, China. We observed geographic variation in the diversity, composition, and functional profiles of dominant mycosphere soil bacterial communities. Soil organic carbon, available potassium, altitude, longitude, and latitude were the key factors affecting mycosphere soil bacterial community structure. Distance–decay analysis revealed that the beta diversity of mycosphere soil bacterial communities declined with increasing geographical distance. Bacterial diversity in the *L. giganteus* mycosphere was significantly lower than that in bulk soil. Genera such as *Micromonospora*, *Streptosporangium*, *Paraburkholderia*, *Pedobacter*, *Caballeronia*, *Paenibacillus*, and *Flavobacterium* could act as saprotrophic helper bacteria in the mycosphere of *L. giganteus*. LEfSe analysis revealed 72 genera that exhibited significant abundance differences among samples from six geographic regions, including *Bradyrhizobium*, *Sphingomicrobium*, and *Phyllobacterium*. Functional annotation detected enriched pathways for transport and catabolism, signal transduction, and carbohydrate and lipid metabolism in mycosphere soil bacterial communities. These results advance our understanding of the ecological interactions of *L. giganteus* and support its artificial cultivation and sustainable resource use.

## 1. Introduction

Soil bacteria are integral to biogeochemical cycling and play critical roles in diverse ecological processes [1]. Bacteria promote hyphal branching by secreting auxin [2], release organic acids that activate phosphatases and enhance litter decomposition [3], and improve the mineralization efficiency of recalcitrant organic compounds, including lignin and cellulose [4]. Beyond nutrient cycling, bacteria participate in the biology of fungi by suppressing pathogens [5], enhancing spore dispersal [6], producing phytohormones and vitamins [7], and increasing fruiting output by reshaping the soil microbial community composition [8]. Soil microbial communities facilitate fruiting body development in edible fungi cultivation and hold promise as microbial inoculants [9,10].

*Leucopaxillus giganteus* (Agaricales: Tricholomataceae), commonly known as the giant funnel or giant leucopax, is a wild basidiomycete distributed across Eurasia and North America [11]. *Leucopaxillus giganteus* exhibits slow growth, which creates substantial obstacles for artificial domestication and cultivation [12]. Modifying medium formulations [13,14] combined with liquid fermentation [15] can accelerate mycelial growth, shorten the spawn production cycle, and improve spawn quality. Harada et al. [11] buried 40 kg of culture medium at a depth of 1 m under *Cryptomeria japonica* forests. *L. giganteus* fruiting bodies subsequently appeared in a fairy ring, and fruiting persisted for five years following inoculation [11]. Its large-sized fruiting bodies have favorable sensory attributes and are rich in nutrients, containing two purified homogeneous polysaccharides: a novel cold-water-soluble polysaccharide (LGP) [16] and a homogeneous low-molecular-weight polysaccharide (DBZG_4-b_) [17]. In contrast, only intracellular and extracellular crude polysaccharides have so far been recovered from its mycelium [18]. In addition to polysaccharides, the fruiting bodies contain proteins, vitamins, 18 amino acids (eight of which are essential amino acids), and ten mineral elements [12,13]. Moreover, *L. giganteus* contains multiple bioactive compounds [16]. Extracts have demonstrated growth-inhibitory activity against HeLa cells and pathogenic bacteria, including *Mycobacterium tuberculosis* and *Brucella abortus* [19,20]. Due to overharvesting and inadequate habitat protection, its wild populations are declining, and its distribution is contracting. Although laboratory cultivation and under forest plantation techniques for *L. giganteus* have been studied, current research still lacks sufficient investigation into its mycosphere soil bacterial communities. Therefore, it is crucial to screen suitable environments for the growth of *L. giganteus*.

In this study, we analyzed bacterial communities in mycosphere and bulk soils of *L. giganteus* from six geographically distinct sites and used PICRUSt2 to predict bacterial functional profiles. We assessed differences in the structure and functional diversity of soil bacterial communities across sampling locations and comprehensively explored suitable growth environments for *L. giganteus* in combination with field habitat conditions. Given the scarce data on bacteria associated with the *L. giganteus* mycosphere, this study sets three primary objectives to aid habitat screening for this mushroom: (1) to identify bacterial biomarker genera in *L. giganteus* mycosphere soil samples from different sites and retrieve taxa potentially associated with favorable growth habitats; (2) to examine how *L. giganteus* affects the diversity and community composition of mycosphere soil bacteria, and improve our understanding of fungus–bacterium interactions in this saprotrophic fungus; and (3) to pinpoint major environmental factors driving mycosphere soil bacterial distributions and provide ecological evidence for *L. giganteus* habitat screening. We accordingly put forward three hypotheses: (1) bacterial biomarkers in the *L. giganteus* mycosphere differ from those of bulk soil and vary across sampling sites; (2) bacterial diversity and community composition within the *L. giganteus* mycosphere show consistent patterns, regardless of spatial differences in background soil conditions; and (3) soil physicochemical properties shape the distribution of mycosphere-associated bacteria and help characterize the environmental needs of *L. giganteus*. The findings are of great significance for the sustainable utilization of *Leucopaxillus giganteus* and the development of its ecological and economic values.

## 2. Materials and Methods

### 2.1. Study Area and Sample Collection

**Figure 1:**
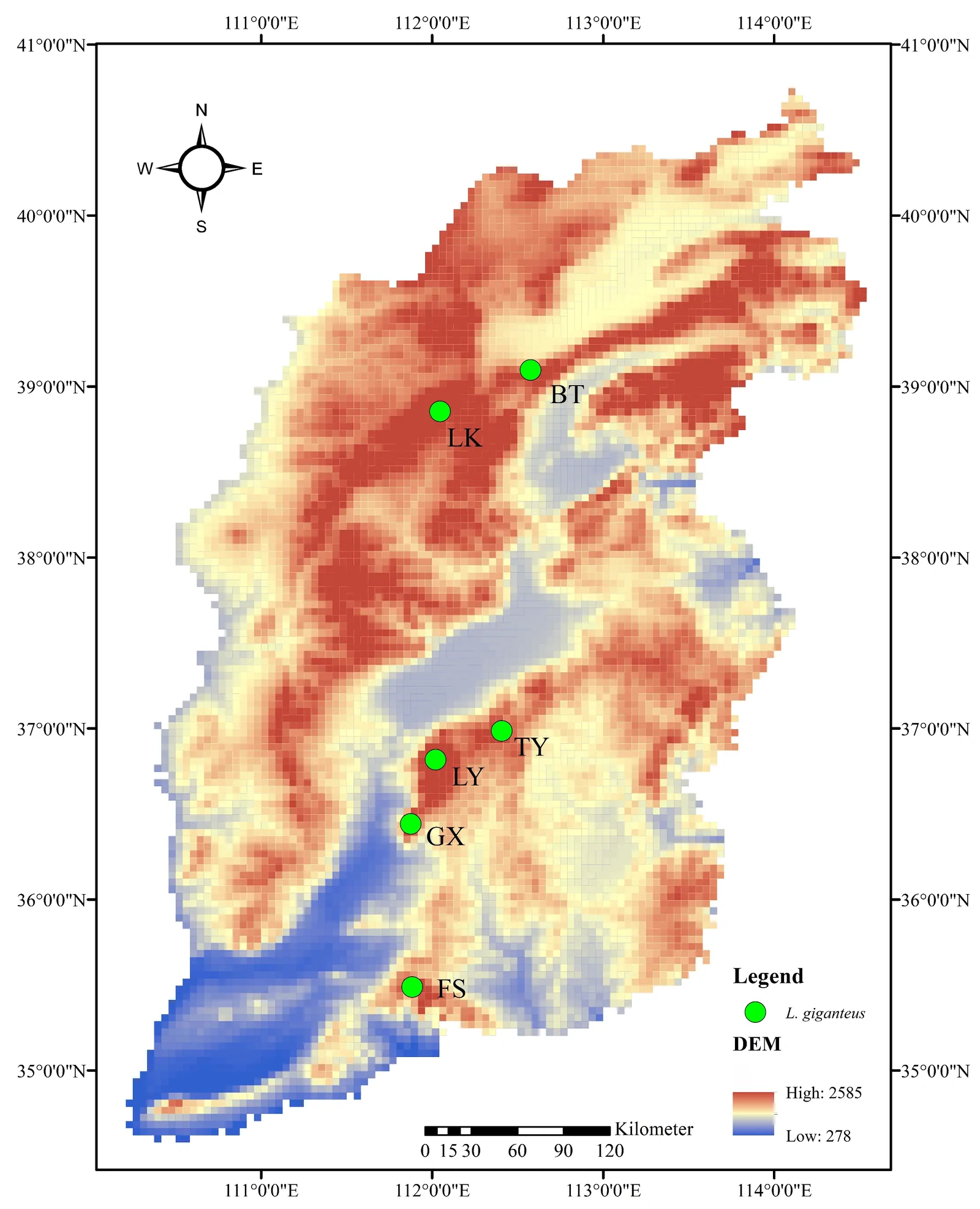
Geographical distribution of sampling sites across Shanxi Province. Mycosphere and bulk soil samples were collected from *Leucopaxillus giganteus* habitats (green circles). DEM, digital elevation model; BT, North Platform of Wutai Mountain; LK, Lingkong Mountain; TY, Taiyue Mountain; LY, Luya Mountain; GX, Gu County; FS, Fushan County. The base map used in this figure was obtained from the Ministry of Natural Resources of the People’s Republic of China (map-approval number GS (2020) 4619), and no modifications were made to the base map.

The study was conducted across Shanxi Province in Northern China (34°34′ N–40°43′ N, 110°14′ E–114°33′ E). During July and August 2024, a total of 36 soil samples were collected from six sites with *L. giganteus* (Figure 1). Each site was located in forested areas with diverse plant species, spanning altitudes of 1652.1–2245.5 m above sea level (Figure 1). The temperature ranged from 22 °C to 26 °C at the time of sample collection. *L. giganteus* was found primarily in mixed forests dominated by *Picea asperata* and *Pinus tabuliformis*. The dominant soil types across these sites were sandy loam brown soil and cinnamon soil.

At each site, three randomly selected *L. giganteus* fruiting bodies spaced at least 20 m apart were excavated to a depth of 10 cm using a sterile trowel. The base of each mushroom fruiting body was collected, the loose surface soil was shaken off, and the thin soil layer adhering to the mushroom base was recovered in sterile polyethylene bags as mycosphere soil [21]. At the same sampling points, soil without visible fungal fruiting bodies was collected 40 cm away from the *L. giganteus* and designated as bulk soil [22,23]. Thus, three mycosphere soil samples paired with three bulk soil samples were collected at each of the six regions. All samples were divided into two subsamples for processing in the laboratory. One portion was naturally air-dried and sieved through a 2 mm mesh to remove debris for analysis of physicochemical properties. The other part was stored in an ultra-low-temperature freezer maintained at −70 °C for subsequent molecular studies.

### 2.2. Soil Physicochemical Analyses

The soil water content (SWC) was determined gravimetrically after drying in an oven at 105 °C for 24 h [24]. Soil pH was measured using a glass electrode in a 1:2.5 soil–water (*w*/*v*) suspension [25]. Soil organic carbon (SOC) was quantified by the potassium dichromate oxidation method with external heating [26]. Available nitrogen (AN) was determined by potassium persulfate oxidation [27], available phosphorus (AP) was determined by the molybdenum–antimony colorimetric method following double-acid extraction [28], and available potassium (AK) was determined by flame photometry [29].

### 2.3. DNA Extraction and 16S rRNA Sequencing

Total genomic DNA was extracted using a modified CTAB protocol [30]. DNA integrity was verified by 1% agarose gel electrophoresis, and purity was confirmed by UV–Vis spectrophotometry with absorbance ratios (A260/A280) between 1.8 and 2.0 and concentrations >50 ng/μL [31]. The bacterial 16S rRNA V3–V4 regions were amplified with the primers 341F (5′-CCTAYGGGRBGCASCAG-3′) and 806R (5′-GGACTACNNGGGTATCTAAT-3′) [32,33]. Each 25 μL PCR reaction contained 15 μL of Phusion^®^ High-Fidelity PCR Master Mix (New England Biolabs, Beijing, China), 2 μL of each primer, 4 μL of template DNA, and 2 μL of ddH_2_O. The thermal cycling parameters were as follows: initial denaturation at 98 °C for 1 min; 30 cycles of 98 °C for 10 s, 50 °C for 30 s, and 72 °C for 30 s; and a final extension at 72 °C for 5 min. Amplification was performed on a Bio-Rad T100 Thermocycler (Hercules, CA, USA). Sequencing libraries were built with the NEBNext^®^ Ultra DNA Library Prep Kit (Illumina, San Diego, CA, USA). After library quality was verified using an Agilent 5400 system (Agilent Technologies, Santa Clara, CA, USA), libraries were sequenced on the Illumina NovaSeq 6000 platform (Illumina, San Diego, CA, USA) to generate 250 bp paired-end reads. The raw reads were deposited in the NCBI Sequence Read Archive (SRA) database (accession number: PRJNA1344482).

### 2.4. Bioinformatics and Community Analyses

Sequence data were imported into QIIME 2 using the import utility. Demultiplexed reads were quality filtered, trimmed, denoised, and merged with the DADA2 plugin, followed by chimera removal to produce amplicon sequence variant (ASV) feature tables [34]. Taxonomic classification was performed using the QIIME 2 feature-classifier plugin against the SILVA v138.2 database (99% similarity threshold) [34]. Based on the obtained ASVs, alpha diversity (Shannon index, Chao1 index, Faith’s phylogenetic diversity index, and observed OTUs) [35] and beta diversity were calculated in QIIME 2, with weighted UniFrac distances used for beta diversity assessment [36]. PICRUSt2 was applied to predict microbial community metabolic pathways annotated against Kyoto Encyclopedia of Genes and Genomes (KEGG) Orthologs [37].

### 2.5. Statistical Analyses

Statistical analyses were conducted on the normally distributed 16S sequencing data of mycosphere and bulk soils. Since variances differed significantly between groups, Welch’s *t*-tests were used for pairwise comparisons [38]. For comparisons among the six regions, intergroup differences in diversity indices were tested using one-way analysis of variance (ANOVA) [39].

Bipartite association network analysis was performed in R v4.2.3 using tidyr v1.3.0, edgeR v3.36.0, indicspecies v1.7.15 and igraph v1.2.11 packages for data processing and preliminary visualization [40]. Taxonomic annotations at different levels of each OTU were separated, and low-abundance taxa were removed. The OTU abundance matrix was normalized using the trimmed mean of M-values (TMM) method to obtain counts per million (CPM) values. Indicator species analysis was conducted with 9999 permutations, and OTUs with *p* < 0.05 were identified as significant indicator taxa. Sample groups and significant indicator OTUs were defined as two sets of nodes to build the bipartite network. The generated node and edge files were imported into Gephi v0.10.1, and the network was visualized using the Fruchterman–Reingold layout [41].

LEfSe (linear discriminant analysis (LDA) effect size) was adopted to screen genus-level bacterial biomarkers with significant intergroup disparities across the six mycosphere sampling sites (LDA score > 2, *p* < 0.05). This was carried out online using the Wekemo Bioincloud platform [42].

Ordination analyses were performed to clarify relationships between bacterial taxa, sample distributions, and environmental factors. Given that detrended correspondence analysis (DCA) showed first axis gradient lengths lower than 3.0, redundancy analysis (RDA) was performed [43]. The ordinary least squares regression was used to fit the correlation curve between geographical distance and bacterial community similarity [44].

Graphical outputs were generated using SPSS v27.0 and Origin v2024 [23]. Spearman correlation analyses were performed to evaluate correlations between bacterial community and environmental variables.

## 3. Results

### 3.1. Sampling of Mycosphere and Bulk Soils

Soil physicochemical properties varied widely among the 36 samples (Table 1). Soil properties differed greatly among the six sites, with mycosphere soil pH varying from 4.58 to 5.98. Mycosphere soils generally had lower pH than their corresponding bulk soils, except at the LK site. This pattern is likely associated with organic-acid release from fungal hyphae. We found significantly higher available nitrogen (AN) concentrations in mycosphere soils at the LK (*p* = 0.034) and TY (*p* = 0.026) sites, whereas AN levels were lower in mycosphere soil at the GX site (Table S1). Soil organic carbon (SOC) was greater in mycosphere soils at LY, BT, LK, and FS, yet reduced at TY and GX. Available phosphorus (AP) showed an increasing trend in mycosphere samples for most sites, though these differences were not statistically significant. Soil water content and available potassium displayed no consistent trends across sampling sites. These findings indicate that the mycosphere of *L. giganteus* is enriched in essential nutrients compared with bulk soils.

### 3.2. Bacterial Communities in Mycosphere and Bulk Soil

**Figure 2:**
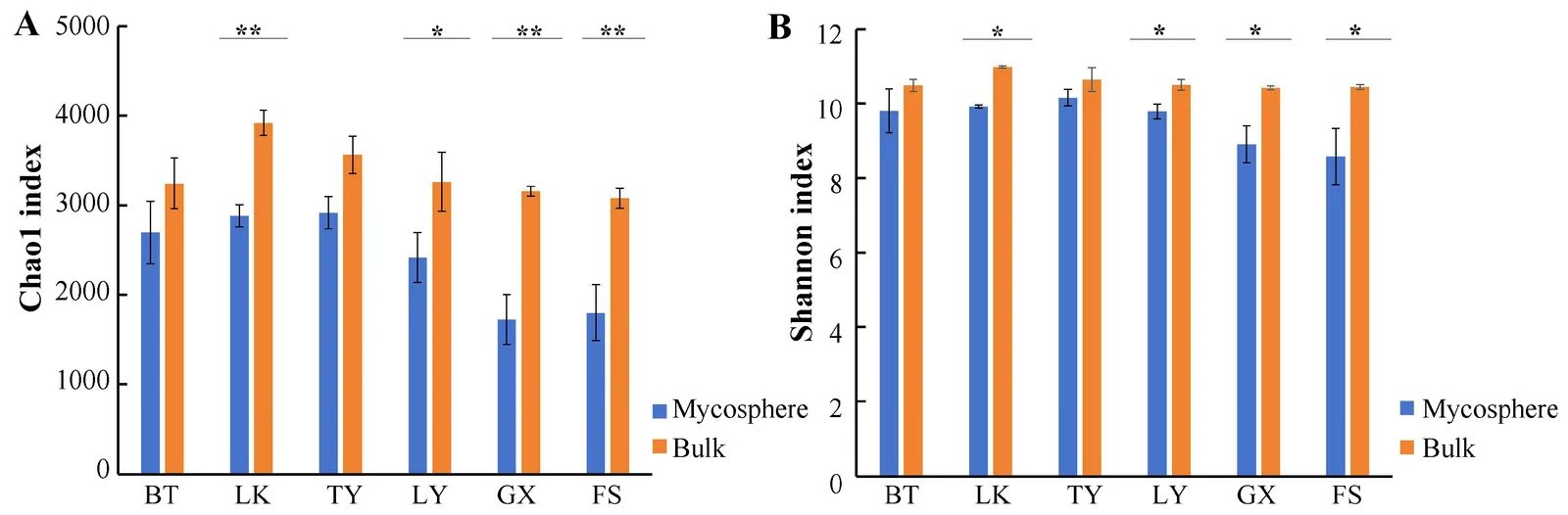
Comparative analysis of the Chao1 index and Shannon index of bacterial communities in mycosphere and bulk soil. (**A**): Chao1 index. (**B**): Shannon index. Data are mean ± SE (*n* = 3). Significant differences by * *p* < 0.05 and ** *p* < 0.01.

After rarefying according to the minimum sample sequence, an average of 45,944 sequences were obtained for each sample for further analysis. The rarefaction curves gradually flattened with increasing sequencing depth, indicating sufficient sequencing coverage and reliable representation of bacterial diversity (Figure S1). The Chao1 index of mycosphere soils from LK, TY, LY, GX, and FS was significantly lower than that of their corresponding bulk soils (*p* < 0.05) (Figure 2A, Table S2). The Shannon index of LK, GX, and FS mycosphere soils was lower (*p* < 0.05) (Figure 2B). Interestingly, Chao1 and Shannon indices were highest in TY mycosphere soils, which showed the highest bacterial abundance and community diversity among all sites (Figure 2).

**Figure 3:**
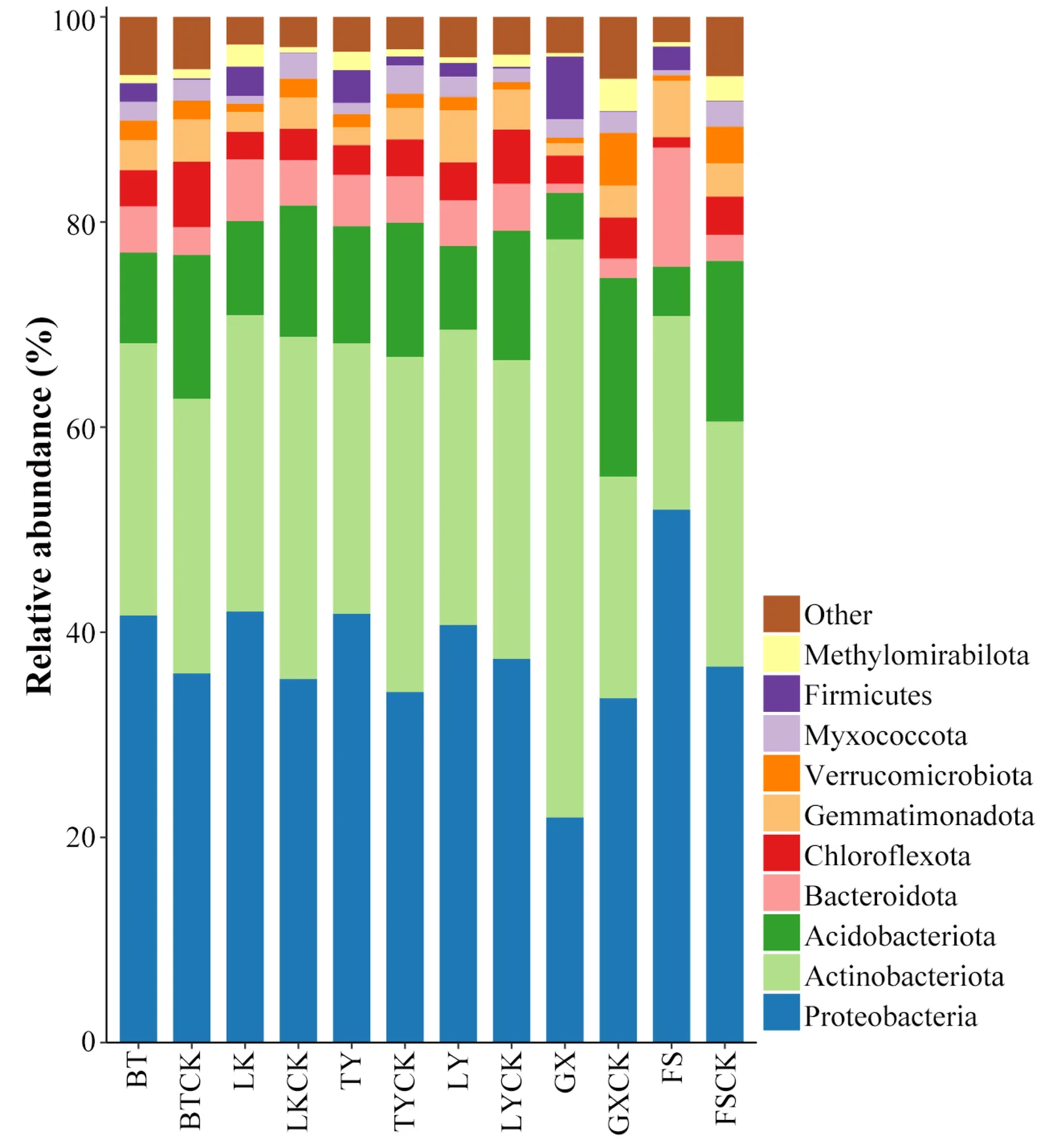
Comparison of phyla between mycosphere and bulk soil. Data are mean (*n* = 3). Soils with the suffix “CK” are bulk soils, while those without “CK” are mycosphere soils.

The 36 soil samples collected from six geographic regions were taxonomically annotated into 45 bacterial phyla. Among these, Proteobacteria, Acidobacteriota, Actinobacteriota, and Bacteroidota were consistently dominant across all samples (Figure 3). At site GX, mycosphere soil showed lower relative abundances of Proteobacteria and higher relative abundances of Actinobacteriota relative to bulk soil; the opposite pattern was observed at the remaining sites (Figure 3). Acidobacteriota exhibited lower relative abundances in mycosphere soil at all six sites (Figure 3). Among the six mycosphere sampling sites, Proteobacteria abundance was significantly lowest at GX and highest at FS (*p* = 0.020) (Table S3).

**Figure 4:**
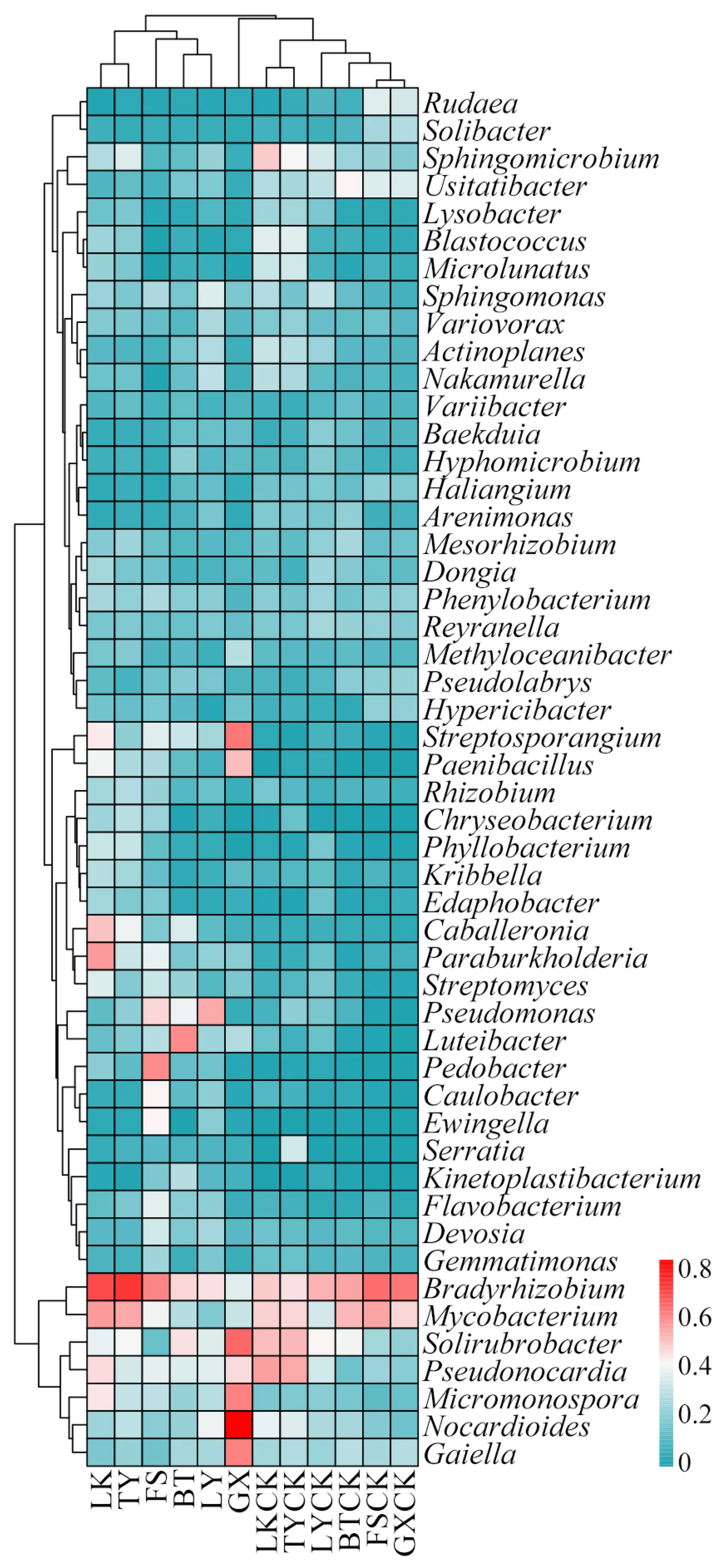
Community composition (top 50 genera) of mycosphere and bulk soil bacteria in six distinct geographic regions. Data are mean (*n* = 3).

A total of 649 bacterial genera were identified across all samples, and further analysis was performed on the top 50 genera (Figure 4). *Bradyrhizobium*, *Mycobacterium*, *Solirubrobacter*, *Nocardioides*, *Pseudonocardia*, *Micromonospora*, *Gaiella*, and *Streptosporangium* represented the dominant genera across sampling sites (Figure 4). Samples from mycosphere soils (LK, TY, FS, BT, LY) displayed greater community variation and specific enrichment of certain taxa. In contrast, bulk soil samples (GXCK, FSCK, BTCK, LYCK, LKCX, TYCX) clustered separately (Figure 4). Within these top 50 genera, nine taxa, namely *Micromonospora*, *Streptosporangium*, *Paraburkholderia*, *Pedobacter*, *Caballeronia*, *Paenibacillus*, *Flavobacterium*, *Chryseobacterium*, and *Kinetoplastibacterium*, exhibited higher relative abundances in mycosphere soils than in bulk soils (Table S4). In contrast, *Sphingomicrobium*, *Usitatibacter*, *Blastococcus*, *Haliangium*, and *Rudaea* were less abundant in mycosphere soils relative to bulk soils (Table S4). Differences in bacterial abundance between mycosphere and bulk soils varied among sampling sites.

At the BT site, *Dongia* (*p* = 0.015) and *Usitatibacter* (*p* = 0.047) showed significantly lower abundance in mycosphere soils compared with bulk soils (Figure 4; Table S4). At the LK site, mycosphere soils exhibited significantly higher abundances of *Caballeronia* (*p* = 0.013), *Micromonospora* (*p* = 0.044), *Paenibacillus* (*p* = 0.0088), and *Pedobacter* (*p* = 0.031); meanwhile, *Nocardioides* (*p* = 0.030), *Pseudonocardia* (*p* = 0.021), *Solirubrobacter* (*p* = 0.018), *Sphingomicrobium* (*p* = 0.00031), and *Usitatibacter* (*p* = 0.0047) were significantly depleted in mycosphere soils. For the TY site, *Caballeronia* (*p* = 0.024), *Hypericibacter* (*p* = 0.036), *Kribbella* (*p* = 0.036), and *Phyllobacterium* (*p* = 0.022) were enriched in mycosphere soils relative to bulk soils, while *Pseudonocardia* (*p* = 0.0090), *Solirubrobacter* (*p* = 0.029), *Microlunatus* (*p* = 0.0017), and *Blastococcus* (*p* = 0.008) were significantly less abundant in mycosphere soils. At the LY site, *Devosia* (*p* = 0.033) was enriched in mycosphere soils, whereas *Blastococcus* (*p* = 0.025) was depleted. Mycosphere soils from the GX site had significantly higher abundances of *Methyloceanibacter* (*p* = 0.00058) and *Paenibacillus* (*p* = 0.015), alongside reduced abundances of *Usitatibacter* (*p* = 0.013), *Phenylobacterium* (*p* = 0.048), and *Solibacter* (*p* = 0.0029). At the FS site, mycosphere soils were enriched in *Rudaea* (*p* < 0.001), *Solibacter* (*p* = 0.0061), *Solirubrobacter* (*p* = 0.048), and *Usitatibacter* (*p* = 0.0016) but depleted in *Blastococcus* (*p* = 0.022), *Haliangium* (*p* = 0.0007), and *Nakamurella* (*p* = 0.041).

**Figure 5:**
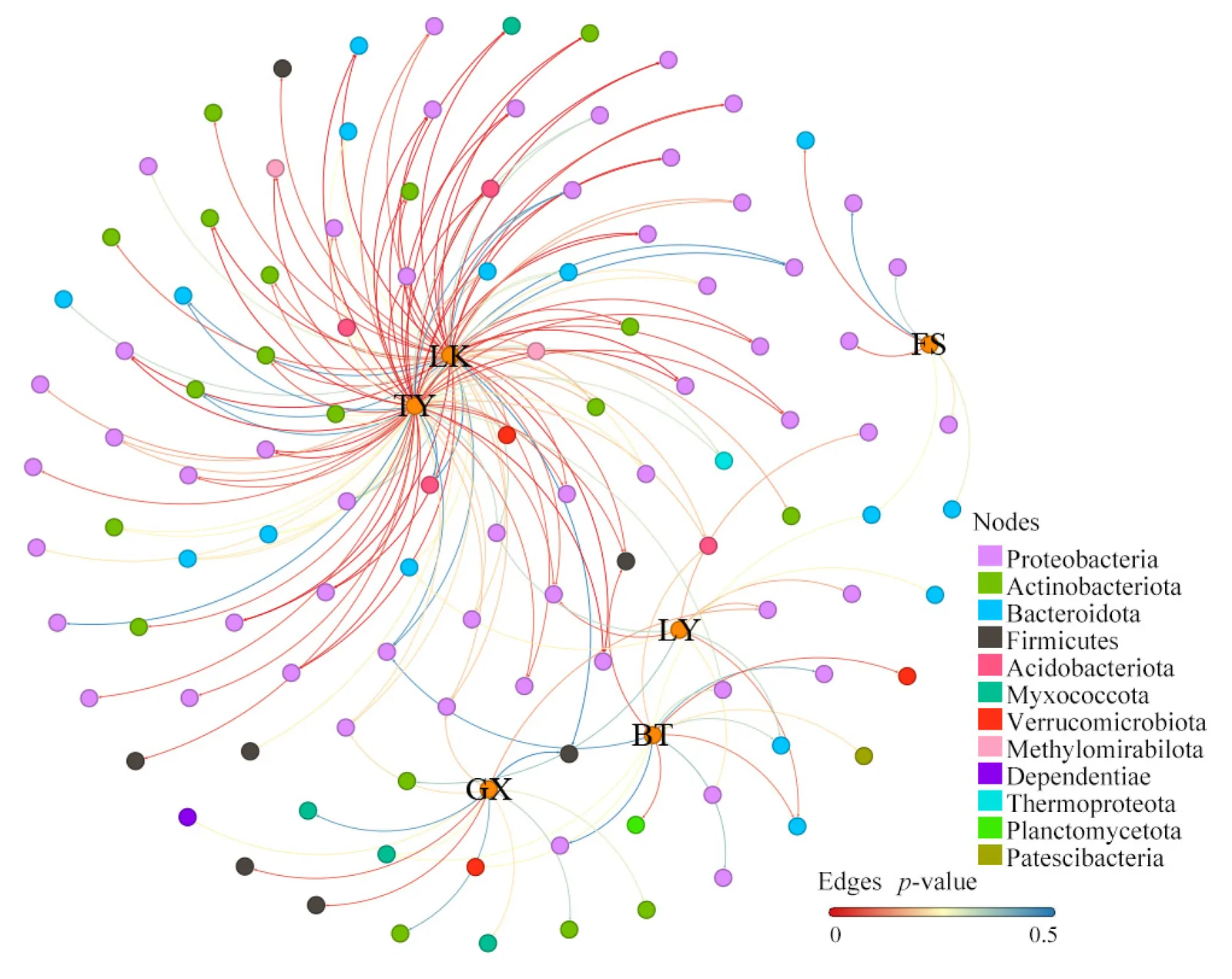
Bipartite network showing associations between sampling sites and indicator OTUs.

Indicator species analysis was performed to construct a bipartite network linking sampling sites and bacterial communities to disentangle community differentiation across sites (Figure 5). The results revealed that the LK and TY sites harbored the richest associated bacterial taxa, with numerous microbial groups showing significant associations with these two sites (Figure 5). In contrast, fewer taxa were associated with FS, LY, BT, and GX. This distribution pattern largely agreed with community diversity analyses based on the top 50 genera (Figure 4). Proteobacteria were ubiquitous across all sampling sites and constituted the shared dominant phylum. Myxococcota, Verrucomicrobiota, and other phyla were only significantly associated with particular sites, exhibiting strong habitat preferences.

**Figure 6:**
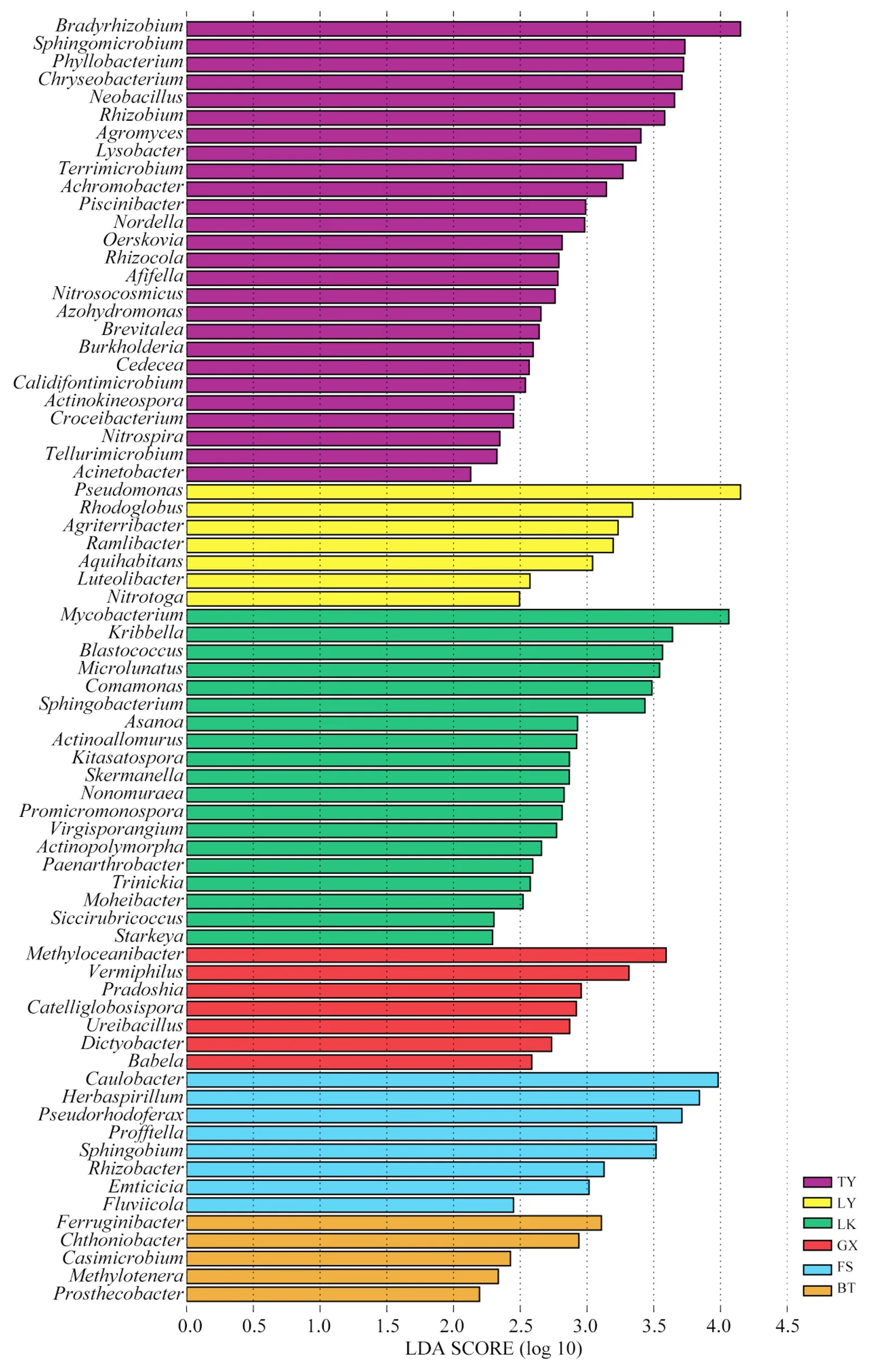
Comparison of bacterial genera across six distinct geographical regions.

LEfSe analysis detected 72 microbial genera with significantly differential abundance across the six geographic regions (BT, FS, GX, LK, LY, and TY) (LDA score > 2, *p* < 0.05) (Figure 6). Genera enriched within each group are presented in the LDA bar chart. The TY group possessed the greatest number of enriched genera (26), including *Bradyrhizobium* (LDA = 4.5), *Sphingomicrobium* (LDA = 4.3), and *Phyllobacterium* (LDA = 4.2). A total of 19 enriched genera were observed in the LK group, among which *Sphingobacterium* and *Comamonas* obtained relatively high LDA values. By comparison, only five enriched genera were screened in the BT group, with *Chthoniobacter* and *Ferruginibacter* serving as the primary biomarkers. The FS group was mainly distinguished by *Profftella* and *Pseudorhodoferax*, whereas *Ureibacillus* and *Catelliglobosispora* were characteristic biomarkers of the GX group. The LY group exhibited prominent enrichment of Pseudomonas (LDA = 4.0) (Figure 6).

### 3.3. Abiotic and Biotic Factors in L. giganteus Mycosphere and Bulk Soils

**Figure 7:**
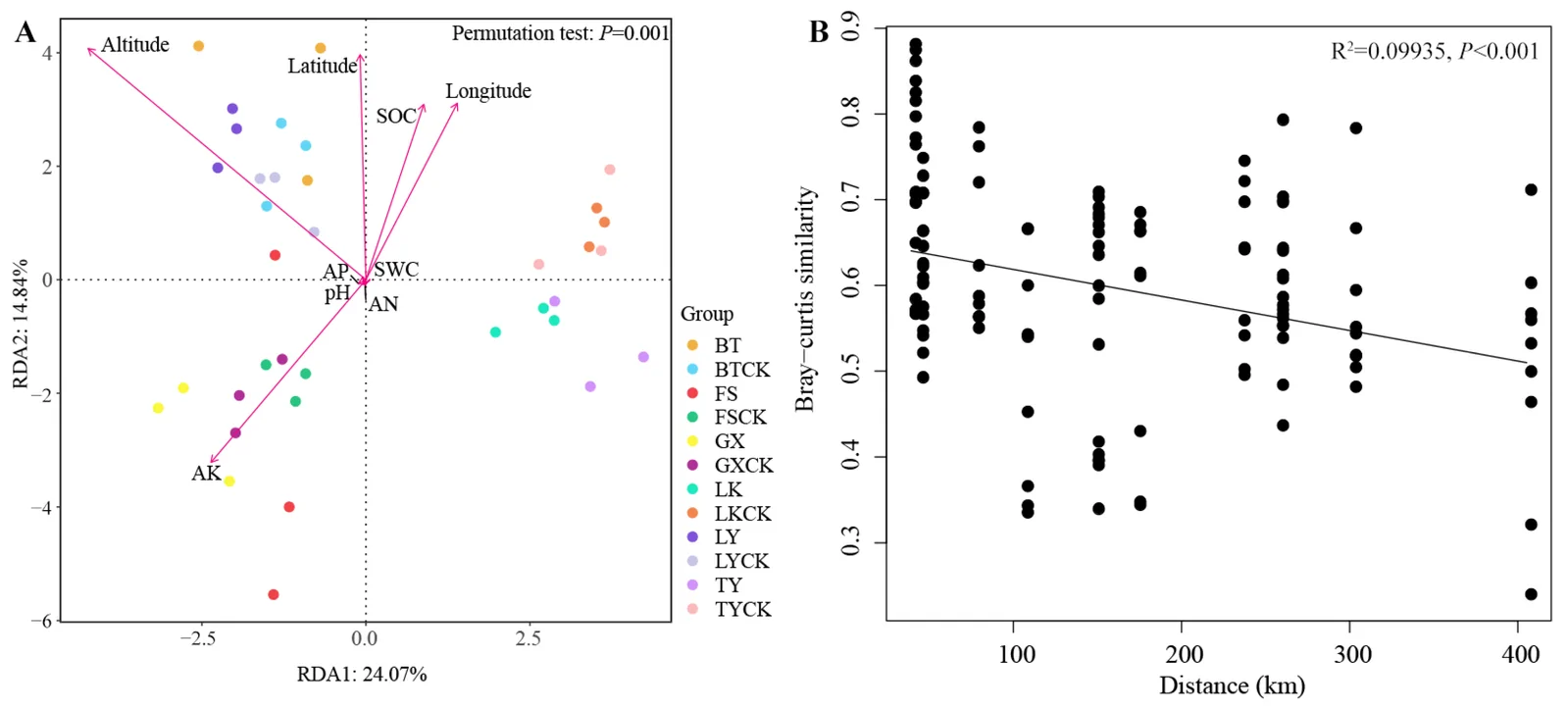
Relationships between bacterial communities and environmental factors. (**A**) Redundancy analysis (RDA) based on bacterial community relative abundances and environmental data. (**B**) Distance–decay patterns for mycosphere soil bacterial communities.

Redundancy analysis (RDA) was used to explore multivariate relationships between bacterial communities, sample distributions, and environmental factors. The first two RDA axes accounted for 24.07% and 14.84% of the total variation in community composition (Figure 7A). SOC (r^2^ = 0.394, *p* = 0.00145), AK (r^2^ = 0.489, *p* = 0.00050), altitude (r^2^ = 0.720, *p* < 0.001), longitude (r^2^ = 0.418, *p* < 0.001), and latitude (r^2^ = 0.487, *p* < 0.001) were identified as key drivers shaping bacterial community assembly. Distance–decay analysis revealed that the beta diversity (Bray–Curtis dissimilarity) of mycosphere soil bacterial communities decreased as geographical distance increased (Figure 7B).

**Figure 8:**
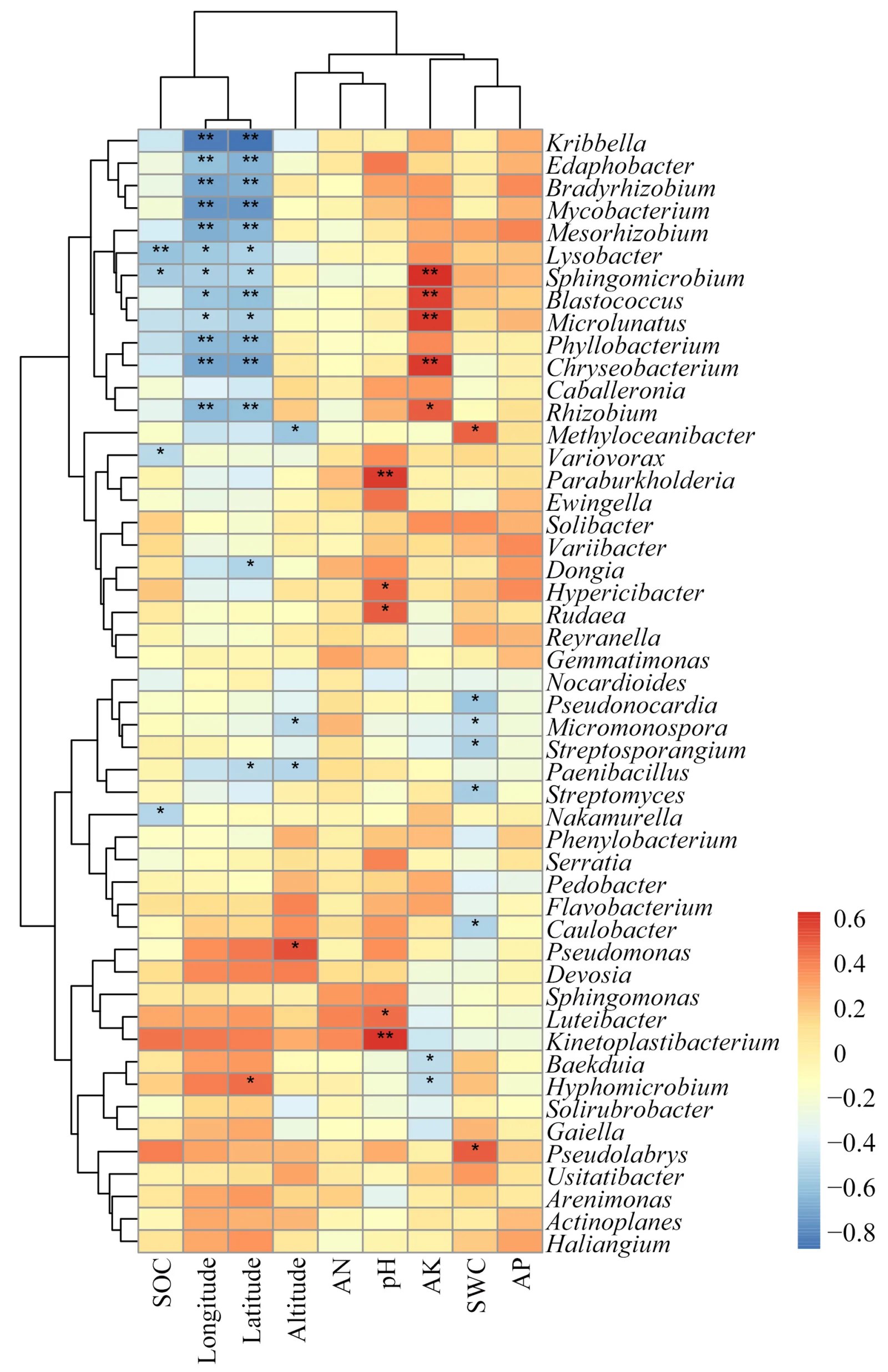
Spearman correlations between the top 50 bacterial genera from mycosphere soils and environmental factors. Significant differences are * *p* < 0.05 and ** *p* < 0.01.

Spearman’s correlation analysis revealed two major clusters of environmental factors: one comprising SOC, longitude and latitude, and another comprising altitude, AN, pH, AK, SWC, and AP (Figure 8). Genus–environment correlations highlighted several significant associations. SWC was positively correlated with *Pseudolabrys* and *Methyloceanibacter* but negatively correlated with *Pseudonocardia*, *Micromonospora*, *Streptosporangium*, and *Streptomyces* (*p* < 0.05). AK showed positive correlations with *Sphingomicrobium*, *Blastococcus*, *Microlunatus*, *Chryseobacterium*, and *Rhizobium* (*p* < 0.05). Soil pH was positively correlated with *Hypericibacter*, *Kinetoplastibacterium*, *Luteibacter*, *Paraburkholderia*, and *Rudaea* (*p* < 0.05). Longitude and latitude were negatively correlated with *Mesorhizobium*, *Bradyrhizobium*, *Kribbella*, *Mycobacterium*, *Edaphobacter*, *Microlunatus*, *Sphingomicrobium*, *Lysobacter*, *Blastococcus*, *Rhizobium*, *Phyllobacterium*, and *Chryseobacterium*. SOC was negatively correlated with *Sphingomicrobium*, *Lysobacter*, *Variovorax*, and *Nakamurella* (*p* < 0.05).

### 3.4. Functional Predictions in Mycosphere and Bulk Soils

**Figure 9:**
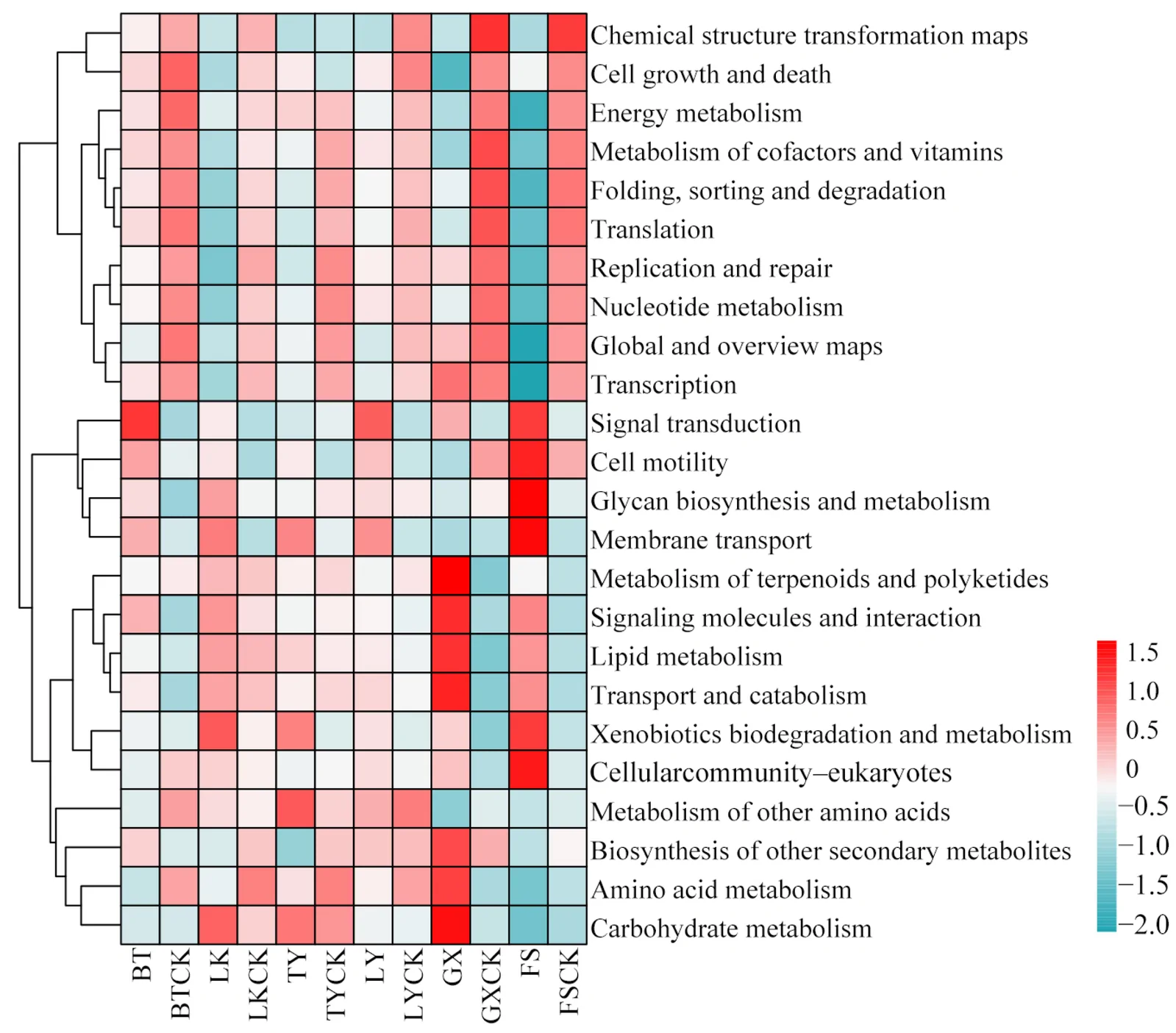
Functional profiles of soil bacterial communities predicted by PICRUSt2 at KEGG level 2.

PICRUSt2 was used to predict functional profiles of soil bacterial communities across the six sampling sites (Figure 9). At KEGG level 2, the clustered heatmap illustrated clear functional differentiation across all samples. Samples belonging to groups BT, LK, TY, and LY harbored higher relative abundances of pathways associated with energy metabolism, translation, replication and repair, and nucleotide metabolism (Figure 9). In contrast, the GX site exhibited higher relative abundances of carbohydrate metabolism, amino acid metabolism, and biosynthesis of other secondary metabolites, while genetic information processing pathways were less abundant (Figure 9). The FS site displayed a distinct functional profile, with elevated abundances of glycan biosynthesis and metabolism as well as chemical structure transformation maps (Figure 9).

Compared with bulk soils, mycosphere soil bacterial communities across the six sites showed higher abundances in cell motility, transport and catabolism, membrane transport, signal transduction, signaling molecules and interactions, carbohydrate metabolism, glycan biosynthesis and metabolism, lipid metabolism, and xenobiotics biodegradation and metabolism. At KEGG level 3, mycosphere soils from all six sites possessed greater abundances of metabolic pathways, such as ABC transporters, nitrogen metabolism, sulfur metabolism, and starch and sucrose metabolism, relative to bulk soils (Table S5).

## 4. Discussion

### 4.1. Regional Variation in Bacterial Community Structure in Mycosphere and Bulk Soil

Previous studies have consistently shown that bacterial diversity is lower in the mycosphere soils of most macrofungi than in bulk soils [45]. Comparable trends occur in other edible fungi, including *Boletus edulis*, *Tylopilus*, *Lactarius hatsudake*, and *Floccularia luteovirens* [46]. We observed the same trend at all six of our sampling sites: both the Chao1 and Shannon indices were lower in mycosphere soil than in bulk soil (Figure 2). Accordingly, the mycosphere of *L. giganteus* supported less bacterial diversity than bulk soil, consistent with previous observations [46,47].

In addition, bacterial richness and community diversity varied among the six geographically distinct sampling sites (Figure 2). This indicates that environmental heterogeneity across sites can modulate the capacity of macrofungi to recruit and filter mycosphere associated bacterial taxa [48]. Harada et al. [11] documented that *Leucopaxillus giganteus* develops dense mycelial mats on the forest floor. The fungus breaks down organic material within *Cryptomeria japonica* stands and alters soil microbial community composition [11]. This indicates that *L. giganteus* is associated with enrichment of growth favoring bacterial taxa and excludes those hampering its development, establishing ecological dominance of certain microbial assemblages. Similar ecological selection has been demonstrated for saprotrophic *Morchella*, where hyphal exudates and microenvironmental changes jointly reshape soil microbial assemblages [23].

The dominant genera in *L. giganteus* mycosphere and bulk soils included *Bradyrhizobium*, *Mycobacterium*, *Solirubrobacter*, *Nocardioides*, *Pseudonocardia*, and *Micromonospora* (Figure 4). Higher relative abundances of *Micromonospora*, *Streptosporangium*, *Pedobacter*, *Paraburkholderia*, *Caballeronia*, *Paenibacillus*, and *Flavobacterium* were detected in mycosphere soils than in bulk soils (Figure 4; Table S4). *Bradyrhizobium*, a Gram-negative alphaproteobacterium, may supply nitrogen to the mycosphere microbiota through nitrogen fixation [49]. *Paraburkholderia* and *Caballeronia* are typical mycosphere adapted bacteria that migrate along fungal hyphae and efficiently utilize hypha-derived organic carbon; they also possess multiple growth promoting capacities, including nitrogen fixation, phosphorus solubilization, and phytohormone secretion, serving as core regulators of fungus–bacterium interactions [50]. Actinobacterial taxa, represented by *Micromonospora*, *Nocardioides*, *Pseudonocardia*, *Streptosporangium*, *Mycobacterium*, and *Solirubrobacter*, degrade recalcitrant substrates, such as chitin and lignin, decompose hyphal residues to drive soil carbon and nitrogen cycling, and produce antimicrobial metabolites to suppress soil borne pathogens and sustain microecological stability [51]. *Pedobacter* also utilizes carbon sources supplied by fungal hyphae while reciprocally releasing nitrogen and phosphorus through decomposition of organic matter, thereby supporting macrofungal development [52]. Collectively, the mycosphere of *L. giganteus* is associated with the enrichment of these functional bacteria, likely via hyphal exudates, which appears to contribute to the assembly of a stable mycosphere bacterial community with functions in nutrient activation and organic decomposition. Meanwhile, allelochemicals from *L. giganteus* appear to correlate with reduced abundance of most soil bacterial taxa, whereas mutualistic or allelochemical-degrading bacteria, such as *Streptosporangium* and *Bradyrhizobium*, show a higher relative abundance within this mycosphere niche [23]. This observed pattern of differential taxon abundance may strengthen the dominance of certain microbial groups, simplify community structure, and reduce overall microbial diversity [9], which provides a plausible explanation for the lower bacterial diversity and richness in mycosphere soil.

*L. giganteus* selectively enriches these functional bacteria via hyphal exudates to establish a stable mycosphere soil bacterial community with functions in nutrient activation, and organic decomposition [53]. Meanwhile, allelochemicals produced by *L. giganteus* broadly inhibit most soil bacterial taxa, while mutualistic or allelochemical degrading bacteria, such as *Streptosporangium* and *Bradyrhizobium*, are resistant or even promoted [23]. This selective filtering strengthens microbial community dominance, simplifies community structure, and reduces overall microbial diversity [9], which explains the lower bacterial diversity and richness in mycosphere soil. Consistent with our observations, related work on saprotrophic fungi *Morchella sextelata* indicates that such host driven filtering is largely governed by homogeneous selection, resulting in relatively resilient bacterial assemblages despite local microenvironmental changes [5].

Analysis of *L. giganteus* bacterial genera across geographic regions revealed that community structure varied among locations, but common dominant taxa were consistently maintained (Figure 4; Table S4). Although significant differences were observed in 72 genera (Figure 6), the majority of genera exhibited no significant differences across sampling sites (Figure 4). Similar patterns have been reported in other macrofungi; for example, convergent microbial diversity, community structure, and functional profiles were observed in *T. matsutake* mycosphere soils across different regions [22]. Geographic variation in the mycosphere soil bacterial diversity of *Agaricus sinodeliciosus* was observed, but shared dominant taxa were maintained across locations [54]. These findings agree with our results, suggesting that while *L. giganteus* mycosphere communities show geographic variability, a core set of dominant helper bacteria persistently co-occurs with this fungus across regions. We recognize that this observation comes from cross-sectional sampling and only demonstrates associations. Although certain genera (e.g., *Micromonospora*, *Streptosporangium*) were consistently more abundant in mycosphere soils (Table S4), further manipulative experiments are needed to verify whether these taxa function as core helper bacteria.

### 4.2. Environmental Factors Affecting the Structure and Composition of Bacterial Community

The growth environment of fungal mycelia modulates both biotic and abiotic components of soil ecosystems [55]. *L. giganteus* is a saprotrophic fungus; its mycelia can interact with plant-root-associated microorganisms and may form facultative mycorrhiza-like associations with plant roots [12,56]. It is found in soils rich in humus within coniferous and mixed forests of broadleaf and conifer trees, where it often produces fairy rings. This key ecological trait among saprotrophic fungi comes from outward mycelial expansion, together with organic matter decomposition [57]. In our study, the AN and AP content were higher in *L. giganteus* mycosphere soils than in bulk soils (Table 1). Saprotrophic fungi secrete laccase, manganese peroxidase, and cellulase to decompose lignin and cellulose in deadwood and leaf litter [58]. They also enrich bacterial genera, such as *Sphingomonas*, *Bradyrhizobium*, and *Pseudomonas*. These bacteria consume cellulose derived oligosaccharides and contribute to overall lignocellulose decomposition within soil microbial communities [23].

Our RDA results demonstrated that soil organic carbon (SOC), available potassium (AK), altitude, longitude, and latitude were the primary abiotic factors influencing *L. giganteus* soil bacterial communities (Figure 7A). In addition to the environmental factors measured in this study, bacterial community assembly is shaped by many unmeasured biotic and abiotic drivers, and residual variation in community composition can stem from neutral stochastic processes, biological interactions, and other undetected environmental conditions [59]. Within ecosystems dominated by saprotrophic fungi, hyphal entanglement physically binds soil particles and improves soil organic carbon (SOC) stability in surface soils [32,60]. Saprotrophic fungi prefer acidic soils, with optimal enzymatic activity and hyphal proliferation occurring at pH 4.0–6.0 [61,62], consistent with the soil pH range (4.79 to 5.98) observed in our study. Soil pH exerts strong deterministic control over bacterial diversity, and saprotrophic fungi shape bacterial community dynamics by mediating pH dependent enzymatic shifts and niche partitioning [62,63]. Available potassium (AK) influences bacterial communities associated with saprotrophic fungi through three interrelated pathways: (i) it directly regulates bacterial potassium uptake systems, (ii) fungi produce indirect regulatory effects as their secretions change and microenvironmental conditions shift, and (iii) functional synergy at the community level enriches taxa antagonistic to pathogens and promotes nutrient cycling [64,65].

Distance–decay analysis showed that the beta diversity (Bray–Curtis dissimilarity) of mycosphere soil bacterial communities declined with increasing geographical distance (Figure 7B), in line with results from Plassart et al. [66]. Driven jointly by climate and edaphic conditions, soil bacteria exhibit obvious biogeographic differentiation, with distinct community compositions across different regions [67]. Collectively, these results suggest that *L. giganteus* can modify soil nutrient status, and soil nutrient conditions may in turn regulate the growth of *L. giganteus*.

### 4.3. Functions of the Bacterial Communities

We performed KEGG functional annotation using PICRUSt2 on soil bacteria from the *L. giganteus* mycosphere. At the KEGG Level 2 pathway, carbohydrate metabolism, cofactor and vitamin metabolism, lipid metabolism, and other amino acid metabolism were among the most abundant functional categories (Figure 9). Earlier studies reported that bacteria rely on metabolic-related pathways to take up amino acids, carbohydrates, vitamins, and other essential nutrients for growth [68].

As a saprotrophic macrofungus, *L. giganteus* exhibits high efficiency in lignin and cellulose degradation. It forms synergistic relationships with heterotrophic bacteria, which substantially improves carbon degradation rates in soil [69]. Root exudates contain carbohydrates, amino acids, fatty acids, and vitamins. These compounds serve as microbial substrates and provide vital carbon sources for soil microbes, driving the enrichment of soil microbial communities [70]. The observed enrichment of nutrient metabolism associated functions indicates that bacteria tend to colonize the *L. giganteus* mycosphere, where nutrients are more accessible [22]. Core functional genes recovered from the mycosphere are not limited to a specific taxonomic group [71]. A set of functional genes displayed higher relative abundances in the mycosphere compared with bulk soil, suggesting that the mycosphere microenvironment imposes selective filtering on these functional traits (Figure 9). Further investigations focused on these functions will improve our understanding of microbial interaction dynamics in *L. giganteus* mycosphere soil.

## 5. Conclusions

We compared the physicochemical properties, bacterial diversity, and community structure of mycosphere and bulk soils across six regions where *L. giganteus* occurs to identify optimal growth conditions. High-throughput 16S rRNA sequencing revealed that the growth of *L. giganteus* significantly altered soil bacterial dynamics, forming a unique mycosphere soil bacterial community with reduced overall diversity and abundance compared to bulk soil. These shifts were primarily driven by the environmental factors SOC, AK, and altitude. The mycosphere enriched key bacterial genera, including *Micromonospora*, *Streptosporangium*, *Paraburkholderia*, *Pedobacter*, and *Caballeronia*, and these taxa serve as potential saprotrophic helper bacteria. Bacterial metabolic pathways involved in immune regulation, membrane transport, and signaling interactions were significantly enriched in the mycosphere compared to bulk soils. Overall, these findings provide new insights into the interactions between *L. giganteus* and its mycosphere-associated bacteria. Accordingly, field management practices including potassium fertilizer application and exogenous organic carbon input, as well as microbial fertilizers containing bacteria that promote *L. giganteus* growth, can aid population propagation and sustainable resource utilization of *L. giganteus*. These potential helper bacterial taxa were screened by comparing community differences between mycosphere and bulk soils. In vitro co-culture and field inoculation trials are required to validate their actual growth-promoting effects on *Leucopaxillus giganteus*.

## Figures and Tables

**Table 1:** Sample site information of soils.

Sample	Longitude (E)	Latitude (N)	Altitude (m)	Vegetation	SWC	pH	SOC (g/kg)	AN (mg/kg)	AP (mg/kg)	AK (mg/kg)
BT	112°34′29.65″	39°5′49.72″	2245.5	*Picea asperata*, *Pinus tabuliformis*	0.49 ± 0.10	4.79 ± 0.64	54.33 ± 1.53	323.41 ± 15.78	2.53 ± 0.59	167.29 ± 9.88
BTCK					0.38 ± 0.03	5.61 ± 0.31	52.66 ± 2.01	335.07 ± 33.65	2.99 ± 0.31	197.72 ± 7.13
LK	112°1′10.59″	36°49′4.46″	1652.1	*Pinus tabuliformis*	0.52 ± 0.10	5.38 ± 0.70	47.61 ± 3.28	400.41 ± 30.56	2.58 ± 0.11	106.55 ± 4.31
LKCK					0.44 ± 0.11	4.58 ± 0.23	46.81 ± 2.21	269.97 ± 27.74	2.11 ± 0.12	125.70 ± 16.14
TY	112°24′22.49″	36°59′10.30″	1767.2	*Pinus tabuliformis*	0.37 ± 0.02	5.98 ± 0.21	43.09 ± 0.77	397.61 ± 16.68	2.96 ± 0.13	173.23 ± 9.60
TYCK					0.38 ± 0.07	6.03 ± 0.13	46.82 ± 1.18	336.01 ± 6.16	2.52 ± 0.26	161.28 ± 4.59
LY	112°2′45.90″	38°51′16.80″	2131.7	*Pinus tabuliformis*	0.41 ± 0.04	5.69 ± 0.17	48.67 ± 0.61	334.37 ± 13.92	3.08 ± 0.27	138.79 ± 21.70
LYCK					0.44 ± 0.01	5.82 ± 0.07	44.68 ± 1.48	309.40 ± 32.65	2.88 ± 0.18	185.48 ± 4.40
GX	111°52′28.04″	36°26′32.76″	1978.9	*Picea asperata*, *Pinus tabuliformis*	0.46 ± 0.14	5.16 ± 0.49	29.26 ± 1.26	307.77 ± 20.03	3.01 ± 0.94	244.49 ± 7.16
GXCK					0.39 ± 0.10	5.59 ± 0.59	31.98 ± 6.18	419.07 ± 13.74	2.65 ± 0.59	235.98 ± 28.04
FS	111°52′56.99″	35°29′17.38″	1688.9	*Pinus tabuliformis*	0.43 ± 0.02	5.63 ± 0.47	43.62 ± 0.61	359.57 ± 14.6	3.26 ± 0.30	232.85 ± 7.19
FSCK					0.46 ± 0.09	5.89 ± 0.49	37.37 ± 5.79	334.61 ± 14.22	2.55 ± 0.24	190.51 ± 16.39

SWC, SOC, AN, AP, and AK represent soil moisture content, soil organic carbon, available nitrogen, available phosphorus, and available potassium, respectively. Data are mean ± standard error (*n* = 3). Soils with the suffix “CK” are bulk soils, while those without “CK” are mycosphere soils.

## Data Availability

The raw fastq file is stored in the NCBI Sequence Read Archive (SRA) database (Accession No: PRJNA1344482).

## Supplementary Materials

The following supporting information can be downloaded at https://www.mdpi.com/article/10.3390/microorganisms14092029/s1, Figure S1: Rarefaction curves of bacterial sequences; Table S1: Comparison of soil physicochemical properties; Table S2: Comparison of bacterial community diversity indices; Table S3: Comparison of soil bacterial communities at the phylum level; Table S4: Welch’s *t*-test comparing mycosphere soil and bulk soil for the top 50 genera; Table S5: Welch’s *t*-test comparing mycosphere soil and bulk soil for KEGG function level 3.
